# Unexpected formation of a co-crystal containing the chalcone (*E*)-1-(5-chloro­thio­phen-2-yl)-3-(3-methyl­thio­phen-2-yl)prop-2-en-1-one and the keto–enol tautomer (*Z*)-1-(5-chloro­thio­phen-2-yl)-3-(3-methyl­thio­phen-2-yl)prop-1-en-1-ol

**DOI:** 10.1107/S2056989020002583

**Published:** 2020-03-03

**Authors:** Mahmoud Al-Refai, Basem F. Ali, Armin Geyer, Klaus Harms, Michael Marsch

**Affiliations:** a Department of Chemistry, Al al-Bayt University, Mafraq 25113, Jordan; b Faculty of Chemistry, Philipps University Marburg, Hans-Meerwein-Strasse 4, 35032, Marburg, Germany

**Keywords:** crystal structure, chalcones, thio­phene, superposition mol­ecular structures

## Abstract

The title crystal structure is assembled from the superposition of two mol­ecular structures, (*E*)-1-(5-chloro­thio­phen-2-yl)-3-(3-methyl­thio­phen-2-yl)prop-2-en-1-one, C_12_H_9_ClOS_2_ (93%), and (*Z*)-1-(5-chloro­thio­phen-2-yl)-3-(3-methyl­thio­phen-2-yl)prop-1-en-1-ol, C_12_H_11_ClOS_2_ (7%), 0.93C_12_H_9_ClOS_2_·0.07C_12_H_11_ClOS_2_. Both were obtained from the reaction of 3-methyl­thio­phene-2-carbaldehyde and 1-(5-chloro­thio­phen-2-yl)ethanone. In the extended structure of the major chalcone component, mol­ecules are linked by a combination of C—H⋯O/S, Cl⋯Cl, Cl⋯π and π–π inter­actions, leading to a compact three-dimensional supra­molecular assembly.

## Abstract 2

The crystallization of (*E*)-1-(5-chloro­thio­phen-2-yl)-3-(3-methyl­thio­phen-2-yl)prop-2-en-1-one furnished a superimposed co-crystal consisting of the expected chalcone and a minor component identified as (*Z*)-1-(5-chloro­thio­phen-2-yl)-3-(3-methyl­thio­phen-2-yl)prop-1-en-1-ol tautomer

## Chemical context   

Chalcones exhibit a wide spectrum of pharmacological activities, including anti­bacterial (Tran *et al.*, 2012 ▸), anti­cancer (Shin *et al.*, 2013 ▸), anti­fungal (López *et al.*, 2001 ▸) and anti-inflammatory properties (Fang *et al.*, 2015 ▸). On the other hand, thio­phene derivatives display a wide range of biological activities such as anti­microbial (Mishra *et al.*, 2012 ▸), anti­allergic (Gillespie *et al.*, 1985 ▸), anti-inflammatory (Molvi *et al.*, 2007 ▸), anti­oxidant and anti­tumor agents (Jarak *et al.*, 2005 ▸). Combining thio­phenes and chalcones could result in compounds with inter­esting new structures and properties: Al-Maqtari *et al.* (2015 ▸) reported the synthesis of thio­phene–chalcones containing two thio­phene rings and their anti­microbial and anti­cancer activities. One of their reported structures is (*E*)-1-(5-chloro­thio­phen-2-yl)-3-(3-methyl­thio­phen-2-yl)prop-2-en-1-one. However, the crystal structure of this thio­phene-based chalcone has not yet been determined.
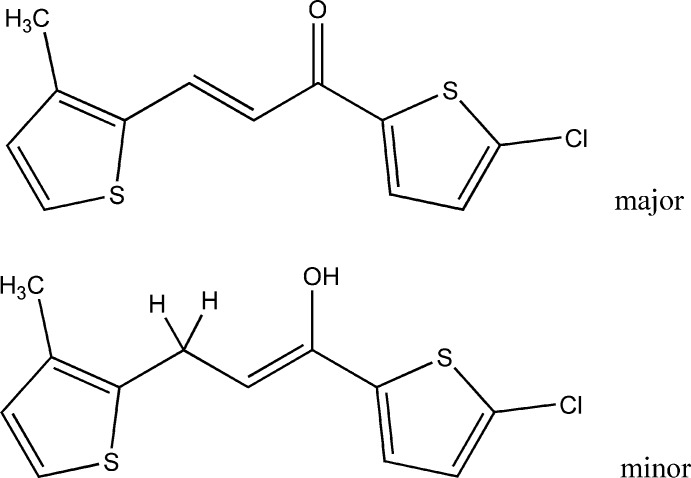


As a part of our ongoing research in this area (Ibrahim *et al.*, 2019 ▸), we report herein the crystal structure of a chalcone containing two terminal-substituted thio­phene rings, namely (*E*)-1-(5-chloro­thio­phen-2-yl)-3-(3-methyl­thio­phen-2-yl)prop-2-en-1-one, which crystallized as a co-crystal in an unexpected superposition with the keto–enol tautomer (*Z*)-1-(5-chloro­thio­phen-2-yl)-3-(3-methyl­thio­phen-2-yl)prop-1-en-1-ol as a minor component.

## Structural commentary   

**Figure 1:**
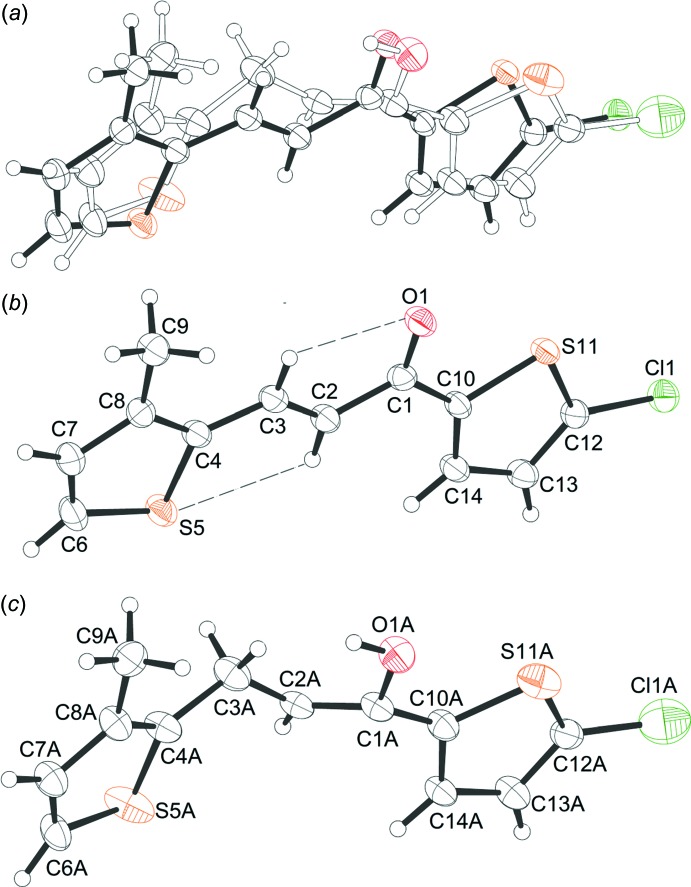
(*a*) The mol­ecular structure of the title co-crystal showing the superposition of the two components, whose occupancies are 93% (black bonds) and 7% (white bonds), (*b*) the mol­ecular structure with the atom-labelling scheme of the major component and (*c*) the minor component. Displacement ellipsoids are drawn at the 50% probability level.

**Figure 2:**
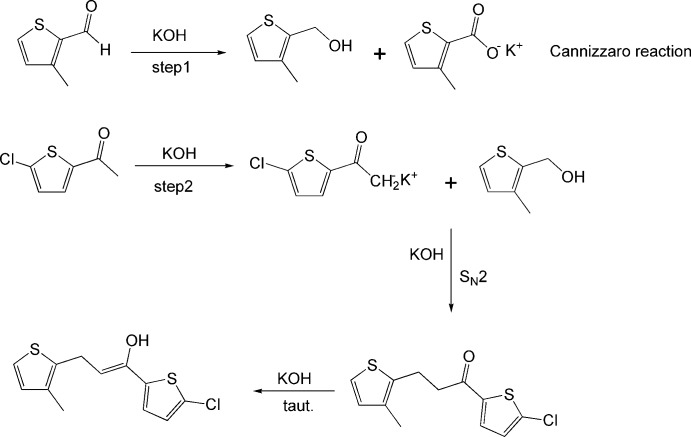
Possible mechanism for the formation of the minor enol component.

The crystal structure (Fig. 1 ▸) exhibits two superimposed mol­ecules with occupancies of 93% and 7%: this was surprising since the formation of the minor (enol) component was quite unexpected. A possible mechanism for the formation of this component is shown in Fig. 2 ▸. Equilibria between keto and enol isomers are regularly observed in solution but not in crystals. This issue needs a thorough exploration, which is beyond the scope of this report.

The mol­ecular structures show similar conformations but differ in bond lengths and the carbon-atom geometry (hybridization), which we will describe for the major component in more detail. The mol­ecular structure (Fig. 1 ▸) is composed of two substituted thio­phene rings, 5-chloro­thio­phen-2-yl and 3-methyl­thio­phen-2-yl, which are linked by the central –CO—CH=CH– spacer. The configuration about the C=C bond [1.344 (3) Å] is *E* and the carbonyl group is *syn* with respect to the C=C bond. The mol­ecule is effectively planar as indicated by the torsion angles O1—C1—C10—C14 = 175.0 (3), C2—C1—C10—C14 = −5.2 (3), C10—C1—C2—C3 = 176.41 (19), O1—C1—C2—C3 = −3.8 (3), C1—C2—C3—C4 = 179.37 (19) and C2—C3—C4—C8 = −177.5 (2)°. The hydrogen atoms of the propenone unit are *trans* configured and each is involved in an intra­molecular short contact that forms an *S*(5) motif (Fig. 1 ▸, Table 1 ▸). The bond lengths and angles are consistent with those in related structures (Vu Quoc *et al.*, 2019 ▸; Yesilyurt *et al.*, 2018 ▸; Sreenatha *et al.*, 2018 ▸). The S atoms of the terminal 5-chloro­thio­phen-2-yl (S11/C10/C12–C14) and 3-methyl­thio­phen-2-yl (S5/C4/C6–C8) rings are *anti* and the rings are inclined slightly to each other [dihedral angle = 6.92 (13)°].

## Supra­molecular features   

**Figure 3:**
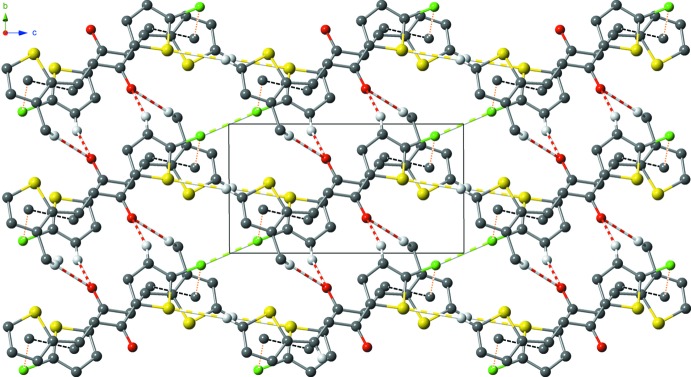
Overall packing of the major component with all inter­molecular inter­actions (dotted and dashed lines) shown.

The extended structure exhibits several hydrogen-bonding contacts (Table 1 ▸). The hydrogen bonds involve a carbonyl O atom serving as a double-acceptor with H atoms from the chloro­thio­phenyl unit, and a methyl group from the methyl­thio­phenyl unit of a neighbouring mol­ecule. Additional C—H⋯S contacts are also present (Table 1 ▸). Further inter­actions are detected, namely Cl⋯Cl [C12—Cl1⋯Cl1^i^ of 3.3907 (8) Å and 142.92 (8)°; symmetry code: (i) −*x*, 2 − *y*, 2 − *z*], C—Cl⋯π [C12—Cl15⋯*Cg* ^ii^ = 3.6536 (14) Å]; symmetry code: (ii) 1 − *x*, 1 − *y*, 1 − *z*; *Cg*1 is the centroid of the S5/C4/C6–C8 ring] as well as π–π contacts [*Cg*1⋯*Cg*2^iii^ of 4.0139 (15) Å; symmetry code: (iii) −*x*, 1 − *y*, 1 − *z*; *Cg*2 is the centroid of the S11/C10/C12–C14 ring], which connect neighbouring mol­ecules, consolidating a rather compact three-dimensional supra­molecular network (Fig. 3 ▸).

## Database survey   

Similar structures to the title compound (major component) with the same chalcone skeleton and one or two thio­phenyl rings include the following, which are identified by their CSD (Groom *et al.*, 2016 ▸) reference codes. In all compounds, the mol­ecular skeletons are approximately planar, and have an *E* configuration about the C=C bond.

The structures containing one thio­phenyl rings include: 1-(5-chloro-2-thien­yl)-3-(2,3,4-tri­meth­oxy­phen­yl)prop-2-en-1-one (refcode LOVHAH; Chidan Kumar *et al.*, 2015 ▸), where the mol­ecular structure features intra­molecular C—H⋯O inter­actions. The mol­ecules in 1-(4-bromo­phen­yl)-3-(3-meth­yl-2-thien­yl)prop-2-en-1-one (XICNON; Fun *et al.*, 2007 ▸), feature short intra­molecular C—H⋯O/S contacts, which form *S*(5) rings. In the crystal structure, the mol­ecules are linked into layers by weak C—H⋯O hydrogen bonds, and short Br⋯O contacts are also observed. In 1-(2-hy­droxy­phen­yl)-3-(5-methyl­thio­phen-2-yl)prop-2-en-1-one (AGE­FUQ; Sreenatha *et al.*, 2018 ▸), the structure exhibits O—H⋯O and C—H⋯O/S intra­molecular inter­actions.

The structures of bis-thio­phenyl chalcones include 2,6-(*E*,*E*)-bis­[(thio­phene-2-yl)methyl­ene]cyclo­hexa­none (BOQ­YAK; Yakalı *et al.*, 2019 ▸) in which the terminal thio­phene rings adopt a *syn* orientation. In the structure, the mol­ecules display weak C—H⋯S and C—H⋯O intra­molecular and only C—H⋯O inter­molecular hydrogen bonds. In addition, π–π inter­actions are found between the thio­phene rings. In 1,5-bis­(3-methyl-2-thien­yl)penta-1,4-dien-3-one (RUZCIZ; Con­treras *et al.*, 2009 ▸), the mol­ecule consists of terminal methyl­thio­phenyl rings with the two S atoms being in a *syn* arrangement and *trans* to the carbonyl oxygen atom. The mol­ecule is almost planar, with a slight twist along the bridging unit, leading to a small rotation between the terminal thio­phenyl rings. The mol­ecules are connected *via* various types of inter­molecular inter­actions, namely C—H⋯O, C—H⋯π and π–π, leading to a three-dimensional supra­molecular network. The mol­ecule of (2*E*,6*E*)-2,6-bis­[(5-methyl­thio­phen-2-yl)methyl­ene]cyclo­hexa­none (XILXUM; Liang *et al.*, 2007 ▸) displays two slightly twisted *syn* terminal methyl­thio­phenyl rings in an *anti*-arrangement with respect to the carbonyl oxygen atom. In 1,5-bis­(thio­phen-3-yl)penta-1,4-dien-3-one (AYUPIU; Shalini *et al.*, 2011 ▸), the dihedral angle between the thio­phenyl rings is 15.45 (10)°. The mol­ecules features both C—H⋯O and C—H⋯π inter­actions. Both thio­phene rings in 3-hy­droxy-1-(thio­phen-2-yl)-3-(thio­phen-3-yl)prop-2-en-1-one (IBIRUJ; Oyarce *et al.*, 2017 ▸) are disordered with the major-disorder components inclined to each other by 12.1 (3)°. In the crystal, the mol­ecules are connected through C—H⋯O inter­actions. In the crystal of 1,3-bis­(3-thien­yl)prop-2-en-1-one (UNAJIE; Baggio *et al.*, 2016 ▸), the thio­phene rings are inclined to each other by a dihedral angle of 8.88 (10)°. The structure exhibits π–π inter­actions together with C—H⋯O inter­actions and short S⋯S contacts also occur.

## Synthesis and crystallization   

The synthesis was carried out using a reported method (Al-Maqtari *et al.*, 2015 ▸). Crystals suitable for single-crystal X-ray diffraction were grown by slow evaporation, at room temperature, of a solution in ethanol.

## Refinement   

Crystal data, data collection and structure refinement details are summarized in Table 2 ▸. Hydrogen atoms were included in calculated positions (C—H = 0.95–0.98 Å) and refined using a riding model with *U*_iso_(H) = 1.2*U*_eq_(C) or 1.5*U*_eq_(C-meth­yl). Methyl groups were allowed to rotate about the bond to their next atom to fit the electron density.

The crystal structure was refined as a superposition of two mol­ecular structures with formulae C_12_H_9_ClOS_2_ (93% occupancy component) and C_12_H_11_ClOS_2_ (7% occupancy component), respectively. Restraints were necessary during the refinement of geometric and anisotropic displacement parameters.

## Supplementary Material

Crystal structure: contains datablock(s) I. DOI: 10.1107/S2056989020002583/hb7894sup1.cif

Structure factors: contains datablock(s) I. DOI: 10.1107/S2056989020002583/hb7894Isup2.hkl

CCDC reference: 1986074

Additional supporting information:  crystallographic information; 3D view; checkCIF report

## Figures and Tables

**Table 1:** Hydrogen-bond geometry (Å, °)

*D*—H⋯*A*	*D*—H	H⋯*A*	*D*⋯*A*	*D*—H⋯*A*
C3—H3⋯O1	0.95	2.48	2.818 (3)	101
C2—H2⋯S5	0.95	2.80	3.166 (2)	104
C13—H13⋯O1^i^	0.95	2.35	3.184 (4)	146
C9—H9*C*⋯O1^ii^	0.98	2.58	3.488 (4)	154
C6—H6⋯S11^iii^	0.95	3.04	3.948 (2)	160

Symmetry codes: (i) 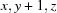; (ii) 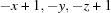; (iii) 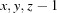.

**Table 2:** Experimental details

Crystal data	
Chemical formula	0.93C_12_H_9_ClOS_2_·0.07C_12_H_11_ClOS_2_
*M*_r_	268.90
Crystal system, space group	Triclinic, *P* 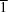
Temperature (K)	100
*a*, *b*, *c* (Å)	7.3709 (4), 7.5063 (4), 12.4247 (6)
α, β, γ (°)	84.126 (4), 76.694 (4), 62.372 (4)
*V* (Å^3^)	592.69 (6)
*Z*	2
Radiation type	Cu *K*α
μ (mm^−1^)	5.93
Crystal size (mm)	0.25 × 0.21 × 0.14

Data collection	
Diffractometer	Stoe Stadivari
Absorption correction	Multi-scan (*LANA*; Stoe, 2016 ▸)
*T*_min_, *T*_max_	0.079, 0.352
No. of measured, independent and observed [*I* > 2σ(*I*)] reflections	10865, 2398, 2221
*R*_int_	0.024
(sin θ/λ)_max_ (Å^−1^)	0.628

Refinement	
*R*[*F* ^2^ > 2σ(*F* ^2^)], *wR*(*F* ^2^), *S*	0.032, 0.088, 1.06
No. of reflections	2398
No. of parameters	291
No. of restraints	754
H-atom treatment	H-atom parameters constrained
Δρ_max_, Δρ_min_ (e Å^−3^)	0.27, −0.28

Computer programs: *X-AREA* (Stoe & Cie, 2016 ▸), *SHELXT2014* (Sheldrick, 2015*a* ▸), *SHELXL2018* (Sheldrick, 2015*b* ▸) and *DIAMOND* (Putz & Brandenburg, 2014 ▸).

## supplementary crystallographic information

### Crystal data

**Table:**

0.93C_12_H_9_ClOS_2_·0.07C_12_H_11_ClOS_2_	*Z* = 2
*M**_r_* = 268.90	*F*(000) = 276
Triclinic, *P*1	*D*_x_ = 1.507 Mg m^−^^3^
*a* = 7.3709 (4) Å	Cu *K*α radiation, λ = 1.54186 Å
*b* = 7.5063 (4) Å	Cell parameters from 17498 reflections
*c* = 12.4247 (6) Å	θ = 3.7–76.0°
α = 84.126 (4)°	µ = 5.93 mm^−^^1^
β = 76.694 (4)°	*T* = 100 K
γ = 62.372 (4)°	Plate, yellow
*V* = 592.69 (6) Å^3^	0.25 × 0.21 × 0.14 mm

### Data collection

**Table:**

Stoe Stadivari diffractometer	2398 independent reflections
Radiation source: GeniX 3D HF Cu	2221 reflections with *I* > 2σ(*I*)
Detector resolution: 5.81 pixels mm^-1^	*R*_int_ = 0.024
rotation method, ω scans	θ_max_ = 75.5°, θ_min_ = 6.7°
Absorption correction: multi-scan (*LANA*; Stoe, 2016)	*h* = −5→9
*T*_min_ = 0.079, *T*_max_ = 0.352	*k* = −8→9
10865 measured reflections	*l* = −13→15

### Refinement

**Table:**

Refinement on *F*^2^	Primary atom site location: dual
Least-squares matrix: full	Secondary atom site location: difference Fourier map
*R*[*F*^2^ > 2σ(*F*^2^)] = 0.032	Hydrogen site location: inferred from neighbouring sites
*wR*(*F*^2^) = 0.088	H-atom parameters constrained
*S* = 1.06	*w* = 1/[σ^2^(*F*_o_^2^) + (0.048*P*)^2^ + 0.4005*P*] where *P* = (*F*_o_^2^ + 2*F*_c_^2^)/3
2398 reflections	(Δ/σ)_max_ < 0.001
291 parameters	Δρ_max_ = 0.27 e Å^−^^3^
754 restraints	Δρ_min_ = −0.28 e Å^−^^3^

### Special details

**Table:**

Geometry. All esds (except the esd in the dihedral angle between two l.s. planes) are estimated using the full covariance matrix. The cell esds are taken into account individually in the estimation of esds in distances, angles and torsion angles; correlations between esds in cell parameters are only used when they are defined by crystal symmetry. An approximate (isotropic) treatment of cell esds is used for estimating esds involving l.s. planes.
Refinement. The crystal structure is an overlay of two molecular structure with ratio 93:7.

### Fractional atomic coordinates and isotropic or equivalent isotropic displacement parameters (Å^2^)

**Table:**

	*x*	*y*	*z*	*U*_iso_*/*U*_eq_	Occ. (<1)
O1	0.3137 (4)	0.2710 (3)	0.5851 (2)	0.0314 (5)	0.93
C1	0.2726 (3)	0.4337 (3)	0.53882 (16)	0.0247 (4)	0.93
C2	0.2535 (3)	0.4652 (3)	0.42238 (15)	0.0259 (4)	0.93
H2	0.213137	0.595628	0.391242	0.031*	0.93
C3	0.2928 (3)	0.3105 (3)	0.35969 (16)	0.0259 (4)	0.93
H3	0.334120	0.182539	0.394094	0.031*	0.93
C4	0.2784 (3)	0.3201 (3)	0.24557 (16)	0.0256 (4)	0.93
S5	0.21123 (9)	0.54296 (8)	0.17199 (4)	0.03242 (16)	0.93
C6	0.2248 (4)	0.4291 (4)	0.05595 (18)	0.0348 (5)	0.93
H6	0.198313	0.496677	−0.011889	0.042*	0.93
C7	0.2777 (4)	0.2310 (3)	0.07204 (18)	0.0314 (4)	0.93
H7	0.291283	0.145132	0.016418	0.038*	0.93
C8	0.3110 (3)	0.1643 (3)	0.18065 (17)	0.0262 (4)	0.93
C9	0.3748 (4)	−0.0483 (3)	0.21906 (19)	0.0312 (5)	0.93
H9A	0.422423	−0.136716	0.154993	0.047*	0.93
H9B	0.254852	−0.057161	0.268618	0.047*	0.93
H9C	0.488738	−0.090123	0.258667	0.047*	0.93
C10	0.2397 (3)	0.6067 (3)	0.60062 (16)	0.0233 (4)	0.93
S11	0.24466 (9)	0.57165 (8)	0.74011 (4)	0.02410 (15)	0.93
C12	0.1984 (3)	0.8151 (3)	0.75171 (17)	0.0251 (4)	0.93
C13	0.1837 (3)	0.9193 (3)	0.65490 (19)	0.0276 (4)	0.93
H13	0.161182	1.054853	0.646974	0.033*	0.93
C14	0.2066 (4)	0.7980 (3)	0.56787 (18)	0.0268 (4)	0.93
H14	0.199724	0.844259	0.494119	0.032*	0.93
Cl1	0.17230 (10)	0.90570 (8)	0.87916 (4)	0.03112 (15)	0.93
O1A	0.354 (6)	0.286 (4)	0.602 (3)	0.035 (6)	0.07
H1A	0.343705	0.215573	0.557340	0.053*	0.07
C1A	0.257 (5)	0.496 (4)	0.569 (2)	0.028 (3)	0.07
C2A	0.126 (4)	0.535 (4)	0.497 (2)	0.029 (3)	0.07
H2A	0.031913	0.671106	0.487244	0.035*	0.07
C3A	0.121 (5)	0.385 (4)	0.435 (2)	0.034 (3)	0.07
H3A	−0.022191	0.397317	0.458163	0.041*	0.07
H3B	0.216209	0.252274	0.460874	0.041*	0.07
C4A	0.175 (5)	0.377 (3)	0.3079 (17)	0.032 (3)	0.07
S5A	0.1043 (15)	0.6046 (12)	0.2374 (7)	0.0485 (18)	0.07
C6A	0.200 (5)	0.482 (3)	0.1107 (16)	0.034 (3)	0.07
H6A	0.209319	0.547565	0.041533	0.041*	0.07
C7A	0.260 (5)	0.281 (3)	0.1218 (18)	0.035 (3)	0.07
H7A	0.296091	0.192728	0.062046	0.042*	0.07
C8A	0.261 (5)	0.219 (3)	0.2331 (19)	0.032 (3)	0.07
C9A	0.331 (5)	0.001 (3)	0.272 (3)	0.036 (5)	0.07
H9AA	0.370744	−0.086268	0.208833	0.053*	0.07
H9AB	0.214815	−0.008058	0.325911	0.053*	0.07
H9AC	0.450522	−0.041072	0.307367	0.053*	0.07
C10A	0.262 (5)	0.615 (4)	0.6395 (18)	0.029 (2)	0.07
S11A	0.3211 (17)	0.5574 (13)	0.7707 (9)	0.0450 (19)	0.07
C12A	0.238 (5)	0.810 (3)	0.7933 (17)	0.031 (3)	0.07
C13A	0.213 (5)	0.918 (4)	0.6986 (19)	0.032 (3)	0.07
H13A	0.186143	1.054308	0.694311	0.039*	0.07
C14A	0.228 (5)	0.812 (4)	0.6075 (19)	0.029 (3)	0.07
H14A	0.217023	0.865457	0.535253	0.034*	0.07
Cl1A	0.255 (2)	0.8778 (18)	0.9186 (11)	0.067 (3)	0.07

### Atomic displacement parameters (Å^2^)

**Table:**

	*U*^11^	*U*^22^	*U*^33^	*U*^12^	*U*^13^	*U*^23^
O1	0.0477 (13)	0.0282 (8)	0.0229 (10)	−0.0213 (8)	−0.0077 (7)	0.0027 (6)
C1	0.0291 (10)	0.0248 (10)	0.0217 (10)	−0.0149 (8)	−0.0016 (8)	−0.0017 (7)
C2	0.0297 (9)	0.0260 (9)	0.0215 (9)	−0.0130 (8)	−0.0042 (7)	0.0013 (7)
C3	0.0287 (10)	0.0281 (9)	0.0216 (9)	−0.0146 (8)	−0.0034 (7)	0.0017 (7)
C4	0.0308 (10)	0.0251 (9)	0.0214 (9)	−0.0141 (8)	−0.0040 (8)	0.0013 (7)
S5	0.0476 (3)	0.0276 (3)	0.0260 (3)	−0.0194 (2)	−0.0118 (2)	0.0051 (2)
C6	0.0465 (12)	0.0405 (12)	0.0214 (9)	−0.0218 (10)	−0.0108 (9)	0.0027 (9)
C7	0.0373 (11)	0.0359 (11)	0.0224 (9)	−0.0177 (9)	−0.0050 (8)	−0.0031 (8)
C8	0.0273 (10)	0.0290 (10)	0.0221 (9)	−0.0132 (8)	−0.0025 (8)	−0.0028 (8)
C9	0.0366 (12)	0.0267 (10)	0.0285 (11)	−0.0132 (9)	−0.0047 (9)	−0.0028 (9)
C10	0.0282 (10)	0.0259 (9)	0.0156 (9)	−0.0130 (7)	−0.0027 (8)	0.0001 (8)
S11	0.0318 (3)	0.0229 (2)	0.0182 (2)	−0.0139 (2)	−0.0033 (2)	0.00121 (18)
C12	0.0315 (10)	0.0249 (9)	0.0207 (9)	−0.0146 (8)	−0.0030 (8)	−0.0036 (8)
C13	0.0327 (11)	0.0245 (9)	0.0252 (10)	−0.0136 (8)	−0.0049 (9)	0.0016 (8)
C14	0.0329 (10)	0.0280 (10)	0.0201 (9)	−0.0154 (8)	−0.0048 (8)	0.0035 (8)
Cl1	0.0378 (3)	0.0319 (3)	0.0246 (3)	−0.0173 (2)	−0.0021 (2)	−0.00631 (19)
O1A	0.045 (11)	0.024 (6)	0.034 (10)	−0.013 (7)	−0.008 (8)	−0.002 (7)
C1A	0.033 (4)	0.027 (4)	0.022 (4)	−0.013 (4)	−0.002 (4)	0.002 (4)
C2A	0.034 (4)	0.028 (4)	0.022 (4)	−0.015 (4)	−0.002 (4)	0.005 (4)
C3A	0.038 (5)	0.030 (5)	0.027 (4)	−0.011 (4)	−0.002 (4)	0.000 (4)
C4A	0.039 (4)	0.028 (4)	0.026 (4)	−0.014 (4)	−0.001 (4)	0.002 (4)
S5A	0.057 (4)	0.035 (3)	0.036 (3)	−0.014 (3)	0.006 (3)	0.005 (3)
C6A	0.047 (4)	0.032 (4)	0.022 (4)	−0.016 (4)	−0.009 (4)	−0.002 (4)
C7A	0.041 (4)	0.035 (4)	0.026 (4)	−0.015 (4)	−0.006 (4)	−0.001 (4)
C8A	0.035 (4)	0.032 (4)	0.025 (4)	−0.012 (4)	−0.005 (4)	0.000 (3)
C9A	0.031 (9)	0.035 (7)	0.039 (10)	−0.014 (7)	−0.007 (9)	−0.002 (7)
C10A	0.034 (4)	0.027 (3)	0.023 (4)	−0.013 (3)	−0.004 (3)	0.000 (3)
S11A	0.045 (4)	0.040 (3)	0.044 (4)	−0.018 (3)	−0.005 (3)	0.008 (3)
C12A	0.036 (4)	0.031 (4)	0.027 (4)	−0.018 (4)	−0.004 (4)	0.001 (4)
C13A	0.038 (5)	0.029 (4)	0.024 (4)	−0.012 (4)	−0.004 (4)	0.001 (4)
C14A	0.035 (4)	0.028 (4)	0.020 (4)	−0.014 (4)	−0.003 (4)	0.002 (4)
Cl1A	0.059 (6)	0.068 (6)	0.070 (7)	−0.030 (5)	−0.004 (5)	−0.001 (5)

### Geometric parameters (Å, º)

**Table:**

O1—C1	1.227 (3)	O1A—H1A	0.8400
C1—C10	1.470 (3)	C1A—C10A	1.328 (10)
C1—C2	1.470 (3)	C1A—C2A	1.38 (4)
C2—C3	1.344 (3)	C2A—C3A	1.44 (4)
C2—H2	0.9500	C2A—H2A	0.9500
C3—C4	1.438 (3)	C3A—C4A	1.54 (3)
C3—H3	0.9500	C3A—H3A	0.9900
C4—C8	1.385 (3)	C3A—H3B	0.9900
C4—S5	1.734 (2)	C4A—C8A	1.386 (18)
S5—C6	1.711 (2)	C4A—S5A	1.744 (16)
C6—C7	1.356 (3)	S5A—C6A	1.734 (18)
C6—H6	0.9500	C6A—C7A	1.367 (18)
C7—C8	1.425 (3)	C6A—H6A	0.9500
C7—H7	0.9500	C7A—C8A	1.414 (18)
C8—C9	1.499 (3)	C7A—H7A	0.9500
C9—H9A	0.9800	C8A—C9A	1.531 (17)
C9—H9B	0.9800	C9A—H9AA	0.9800
C9—H9C	0.9800	C9A—H9AB	0.9800
C10—C14	1.374 (3)	C9A—H9AC	0.9800
C10—S11	1.732 (2)	C10A—C14A	1.412 (18)
S11—C12	1.712 (2)	C10A—S11A	1.743 (18)
C12—C13	1.364 (3)	S11A—C12A	1.735 (17)
C12—Cl1	1.722 (2)	C12A—C13A	1.356 (18)
C13—C14	1.415 (3)	C12A—Cl1A	1.732 (17)
C13—H13	0.9500	C13A—C14A	1.399 (19)
C14—H14	0.9500	C13A—H13A	0.9500
O1A—C1A	1.453 (10)	C14A—H14A	0.9500
			
O1—C1—C10	119.75 (19)	C10A—C1A—O1A	112 (2)
O1—C1—C2	122.7 (2)	C2A—C1A—O1A	113 (2)
C10—C1—C2	117.52 (17)	C1A—C2A—C3A	126 (3)
C3—C2—C1	120.42 (18)	C1A—C2A—H2A	117.2
C3—C2—H2	119.8	C3A—C2A—H2A	117.2
C1—C2—H2	119.8	C2A—C3A—C4A	123 (2)
C2—C3—C4	126.27 (19)	C2A—C3A—H3A	106.7
C2—C3—H3	116.9	C4A—C3A—H3A	106.7
C4—C3—H3	116.9	C2A—C3A—H3B	106.7
C8—C4—C3	127.20 (18)	C4A—C3A—H3B	106.7
C8—C4—S5	111.23 (14)	H3A—C3A—H3B	106.6
C3—C4—S5	121.56 (15)	C8A—C4A—C3A	132.5 (18)
C6—S5—C4	91.72 (10)	C8A—C4A—S5A	110.0 (13)
C7—C6—S5	112.16 (16)	C3A—C4A—S5A	117.4 (16)
C7—C6—H6	123.9	C6A—S5A—C4A	91.4 (10)
S5—C6—H6	123.9	C7A—C6A—S5A	112.2 (15)
C6—C7—C8	113.4 (2)	C7A—C6A—H6A	123.9
C6—C7—H7	123.3	S5A—C6A—H6A	123.9
C8—C7—H7	123.3	C6A—C7A—C8A	111.7 (18)
C4—C8—C7	111.53 (18)	C6A—C7A—H7A	124.2
C4—C8—C9	124.48 (19)	C8A—C7A—H7A	124.2
C7—C8—C9	123.99 (19)	C4A—C8A—C7A	113.9 (16)
C8—C9—H9A	109.5	C4A—C8A—C9A	121.3 (19)
C8—C9—H9B	109.5	C7A—C8A—C9A	125 (2)
H9A—C9—H9B	109.5	C8A—C9A—H9AA	109.5
C8—C9—H9C	109.5	C8A—C9A—H9AB	109.5
H9A—C9—H9C	109.5	H9AA—C9A—H9AB	109.5
H9B—C9—H9C	109.5	C8A—C9A—H9AC	109.5
C14—C10—C1	131.62 (19)	H9AA—C9A—H9AC	109.5
C14—C10—S11	111.50 (16)	H9AB—C9A—H9AC	109.5
C1—C10—S11	116.88 (14)	C1A—C10A—C14A	120 (2)
C12—S11—C10	90.29 (10)	C1A—C10A—S11A	128.3 (19)
C13—C12—S11	114.16 (16)	C14A—C10A—S11A	111.9 (14)
C13—C12—Cl1	126.56 (16)	C12A—S11A—C10A	89.6 (10)
S11—C12—Cl1	119.28 (12)	C13A—C12A—Cl1A	129.0 (16)
C12—C13—C14	110.72 (18)	C13A—C12A—S11A	111.6 (14)
C12—C13—H13	124.6	Cl1A—C12A—S11A	117.7 (12)
C14—C13—H13	124.6	C12A—C13A—C14A	115.1 (17)
C10—C14—C13	113.32 (18)	C12A—C13A—H13A	122.4
C10—C14—H14	123.3	C14A—C13A—H13A	122.4
C13—C14—H14	123.3	C13A—C14A—C10A	109.6 (17)
C1A—O1A—H1A	109.5	C13A—C14A—H14A	125.2
C10A—C1A—C2A	131 (3)	C10A—C14A—H14A	125.2
			
O1—C1—C2—C3	−3.8 (3)	C10A—C1A—C2A—C3A	−171 (3)
C10—C1—C2—C3	176.41 (19)	O1A—C1A—C2A—C3A	−16 (5)
C1—C2—C3—C4	179.37 (19)	C1A—C2A—C3A—C4A	−117 (3)
C2—C3—C4—C8	−177.5 (2)	C2A—C3A—C4A—C8A	150 (3)
C2—C3—C4—S5	1.6 (3)	C2A—C3A—C4A—S5A	−35 (4)
C8—C4—S5—C6	0.42 (17)	C8A—C4A—S5A—C6A	−2 (3)
C3—C4—S5—C6	−178.88 (18)	C3A—C4A—S5A—C6A	−178 (3)
C4—S5—C6—C7	0.04 (19)	C4A—S5A—C6A—C7A	7 (3)
S5—C6—C7—C8	−0.5 (3)	S5A—C6A—C7A—C8A	−10 (4)
C3—C4—C8—C7	178.5 (2)	C3A—C4A—C8A—C7A	172 (3)
S5—C4—C8—C7	−0.7 (2)	S5A—C4A—C8A—C7A	−3 (4)
C3—C4—C8—C9	−1.9 (3)	C3A—C4A—C8A—C9A	−2 (6)
S5—C4—C8—C9	178.87 (17)	S5A—C4A—C8A—C9A	−178 (2)
C6—C7—C8—C4	0.8 (3)	C6A—C7A—C8A—C4A	9 (4)
C6—C7—C8—C9	−178.8 (2)	C6A—C7A—C8A—C9A	−177 (3)
O1—C1—C10—C14	175.0 (3)	C2A—C1A—C10A—C14A	−41 (6)
C2—C1—C10—C14	−5.2 (3)	O1A—C1A—C10A—C14A	163 (3)
O1—C1—C10—S11	−4.2 (3)	C2A—C1A—C10A—S11A	143 (3)
C2—C1—C10—S11	175.62 (15)	O1A—C1A—C10A—S11A	−13 (5)
C14—C10—S11—C12	0.86 (17)	C1A—C10A—S11A—C12A	−170 (4)
C1—C10—S11—C12	−179.83 (17)	C14A—C10A—S11A—C12A	13 (3)
C10—S11—C12—C13	−1.24 (18)	C10A—S11A—C12A—C13A	−12 (3)
C10—S11—C12—Cl1	178.05 (14)	C10A—S11A—C12A—Cl1A	−179 (2)
S11—C12—C13—C14	1.3 (3)	Cl1A—C12A—C13A—C14A	173 (3)
Cl1—C12—C13—C14	−177.96 (16)	S11A—C12A—C13A—C14A	9 (4)
C1—C10—C14—C13	−179.5 (2)	C12A—C13A—C14A—C10A	2 (4)
S11—C10—C14—C13	−0.3 (2)	C1A—C10A—C14A—C13A	172 (3)
C12—C13—C14—C10	−0.6 (3)	S11A—C10A—C14A—C13A	−11 (4)

### Hydrogen-bond geometry (Å, º)

**Table:**

*D*—H···*A*	*D*—H	H···*A*	*D*···*A*	*D*—H···*A*
C3—H3···O1	0.95	2.48	2.818 (3)	101
C2—H2···S5	0.95	2.80	3.166 (2)	104
C13—H13···O1^i^	0.95	2.35	3.184 (4)	146
C9—H9*C*···O1^ii^	0.98	2.58	3.488 (4)	154
C6—H6···S11^iii^	0.95	3.04	3.948 (2)	160

Symmetry codes: (i) *x*, *y*+1, *z*; (ii) −*x*+1, −*y*, −*z*+1; (iii) *x*, *y*, *z*−1.

## References

[bb1] Al-Maqtari, H. M., Jamalis, J. & Sirat, H. M. (2015). *Jurnal Teknologi*, **77**, 55–59.

[bb2] Baggio, R., Brovelli, F., Moreno, Y., Pinto, M. & Soto-Delgado, J. (2016). *J. Mol. Struct.* **1123**, 1–7.

[bb3] Chidan Kumar, C. S., Govindarasu, K., Fun, H.-K., Kavitha, E., Chandraju, S. & Quah, C. K. (2015). *J. Mol. Struct.* **1085**, 63–77.

[bb21] Contreras, D., Moreno, Y., Soto, C., Saavedra, M., Brovelli, F. & Baggio, R. (2009). *J. Chil. Chem. Soc.* **54**, 470–472.

[bb4] Fang, Q., Zhao, L., Wang, Y., Zhang, Y., Li, Z., Pan, Y., Kanchana, K., Wang, J., Tong, C., Li, D. & Liang, G. (2015). *Toxicol. Appl. Pharmacol.* **282**, 129–138. 10.1016/j.taap.2014.10.021 25447405

[bb5] Fun, H.-K., Chantrapromma, S., Patil, P. S. & Dharmaprakash, S. M. (2007). *Acta Cryst.* E**63**, o2724–o2725.

[bb6] Gillespie, E., Dungan, K. W., Gomoll, A. W. & Seidehamel, R. J. (1985). *Int. J. Immunopharmacol.* **7**, 655–660. 10.1016/0192-0561(85)90149-3 2412976

[bb7] Groom, C. R., Bruno, I. J., Lightfoot, M. P. & Ward, S. C. (2016). *Acta Cryst.* B**72**, 171–179. 10.1107/S2052520616003954 PMC4822653 27048719

[bb9] Ibrahim, M. M., Al-Refai, M., Ali, B. F., Geyer, A., Harms, K. & Marsch, M. (2019). *IUCrData*, **4**, x191046.

[bb10] Jarak, I., Kralj, M., Šuman, L., Pavlović, G., Dogan, J., Piantanida, I., Žinić, M., Pavelić, K. & Karminski-Zamola, G. (2005). *J. Med. Chem.* **48**, 2346–2360. 10.1021/jm049541f 15801828

[bb11] Liang, G., Yang, S.-L., Wang, X.-H., Li, Y.-R. & Li, X.-K. (2007). *Acta Cryst.* E**63**, o4118.

[bb12] López, S. N., Castelli, M., Zacchino, S. A., Domínguez, J. N., Lobo, G., Charris-Charris, J., Cortés, J. C. G., Ribas, J. C., Devia, C., Rodríguez, A. M. & Enriz, R. D. (2001). *Bioorg. Med. Chem.* **9**, 1999–2013. 10.1016/s0968-0896(01)00116-x 11504637

[bb13] Mishra, R., Tomer, I. & Kumar, S. (2012). *Der Pharmacia Sinica*, **3**, 332–336.

[bb14] Molvi, K. I., Vasu, K. K., Yerande, S. G., Sudarsanam, V. & Haque, N. (2007). *Eur. J. Med. Chem.* **42**, 1049–1058. 10.1016/j.ejmech.2007.01.007 17336429

[bb15] Oyarce, J., Hernández, L., Ahumada, G., Soto, J. P., del Valle, M. A., Dorcet, V., Carrillo, D., Hamon, J.-R. & Manzur, C. (2017). *Polyhedron*, **123**, 277–284.

[bb16] Putz, H. & Brandenburg, K. (2014). *DIAMOND*. Crystal Impact GbR, Bonn, Germany.

[bb17] Shalini, S., Girija, C. R., Jotani, M. M., Rajashekhar, B., Rao, N. & Tiekink, E. R. T. (2011). *Acta Cryst.* E**67**, o2354. 10.1107/S160053681103248X PMC3200598 22058969

[bb18] Sheldrick, G. M. (2015*a*). *Acta Cryst.* A**71**, 3–8.

[bb19] Sheldrick, G. M. (2015*b*). *Acta Cryst.* C**71**, 3–8.

[bb20] Shin, S. Y., Yoon, H., Hwang, D., Ahn, S., Kim, D.-W., Koh, D., Lee, Y. H. & Lim, Y. (2013). *Bioorg. Med. Chem.* **21**, 7018–7024. 10.1016/j.bmc.2013.09.014 24095020

[bb22] Sreenatha, N. R., Lakshminarayana, B. N., Ganesha, D. P., Vijayshankar, S. & Nagaraju, S. (2018). *X-ray Struct. Anal. Online*, **34**, 23–24.

[bb23] Stoe & Cie (2016). *X-AREA* and *LANA*. Stoe & Cie GmbH, Darmstadt, Germany.

[bb24] Tran, T. D., Nguyen, T. T., Do, T. H., Huynh, T. N., Tran, C. D. & Thai, K. M. (2012). *Molecules*, **17**, 6684–6696. 10.3390/molecules17066684 PMC6268422 22728362

[bb25] Vu Quoc, T., Tran Thi Thuy, D., Dang Thanh, T., Phung Ngoc, T., Nguyen Thien, V., Nguyen Thuy, C. & Van Meervelt, L. (2019). *Acta Cryst.* E**75**, 957–963. 10.1107/S2056989019007503 PMC6659342 31392003

[bb26] Yakalı, G., Biçer, A. & Cin, G. T. (2019). *Turk. C. Theo. Chem.* **3**, 47–58.

[bb27] Yesilyurt, F., Aydin, A., Gul, H. I., Akkurt, M. & Ozcelik, N. D. (2018). *Acta Cryst.* E**74**, 960–963. 10.1107/S2056989018008459 PMC6038634 30002894

